# Optimising ovarian cancer care: An embedded mixed methods evaluation of a national nurse-led telehealth program

**DOI:** 10.1007/s00520-026-11284-8

**Published:** 2026-10-07

**Authors:** Patsy Yates, Wei-Hong Liu, Vanissa Ong, Sue Hegarty, Cyril Dixon, Anna Tart, Natalie Williams, Bridget Bradhurst

**Affiliations:** 1 Cancer and Palliative Care Outcomes Centre, Centre for Healthcare Transformation, Faculty of Health, Queensland University of Technology, Brisbane, QLD Australia; 2 Pancare Foundation, Melbourne, VIC Australia; 3 Melbourne, Victora Australia; 4 Ovarian Cancer Australia, Melbourne, VIC Australia; 5 School of Nursing, Curtin University, Bentley, WA Australia

**Keywords:** Ovarian cancer, Mixed methods study, Telehealth, Patient reported outcome measures, Cancer nursing, Supportive cancer care

## Abstract

**Purpose:**

A nationwide nurse-led telehealth case management program was established providing personalised supportive care to individuals and families experiencing ovarian cancer. The program was evaluated using the RE-AIM framework. This paper focuses on the effectiveness findings of the evaluation.

**Methods:**

Using mixed methods design, participants completed four surveys over 12 months assessing symptom burden, psychological distress, supportive care needs, quality of life, and self-efficacy. Service experiences were further explored through interviews.

**Results:**

Of the 652 individuals enrolled in the program, 112 (17%) participated in the evaluation. Predominant baseline moderate to severe symptoms were fatigue (65%); anxiety (56%); and sleep issues (50%). Key unmet needs included: uncertainty about the future (54%); fear of cancer spread (48%); and anxiety (42%). 43% reported high distress; 27% lacked confidence self-managing fatigue, 23% self-managing distress. All participants received symptom and needs assessment; 80% received information support, and one-third accessed specialised psychosocial care. Those with greater symptom burden, distress, lower quality of life and self-efficacy at baseline received more services. Over 12 months, overall wellbeing and satisfaction with information improved significantly. 30 interview participants consistently valued the program for: being seen and heard as a whole person; trusted information and advice; meaningful emotional support; and flexible and timely services.

**Conclusion:**

Participation in this nurse-led telehealth program was associated with improved outcomes and positive experiences among individuals with ovarian cancer who are at high risk of poorer outcomes. These findings support continued investment in person-centred tailored supportive care delivered via telehealth prioritising emotional wellbeing, symptom management, and quality of life.

**Supplementary Information:**

The online version contains supplementary material available at https://doi.org/10.1007/s00520-026-11284-8.

## Introduction

Ovarian cancers, including fallopian tube and primary peritoneal cancers, are the second most prevalent gynaecological cancer types in Australia, with approximately 1900 new cases estimated in 2025 [1]. Exacerbated by the absence of effective early detection methods, around 75% of cases are diagnosed at an advanced stage, requiring intensive treatment and accompanied by significant side effects. Despite advances in treatment options, most individuals experience recurrence within two years of initial treatment [2, 3], and the five-year survival rate remains below 50% [1]. Beyond clinical outcomes, many studies [4–11] have shown that individuals with ovarian cancer endure substantial and multifaceted burdens in physical, psychological, and social domains, which severely compromise their quality of life. These challenges underscore the critical need for holistic, person-centred models of comprehensive supportive care throughout the cancer experience.

Supportive care is considered a core component of comprehensive patient-centred cancer care to assess and manage the symptoms and needs of patients and their families across the entire cancer experience [12]. In Australia, hospital-based care during and after cancer treatment often results in fragmented and inconsistent ways of addressing complex supportive care needs known to impact individuals with ovarian cancer [13, 14]. In 2017, Ovarian Cancer Australia (OCA), an independent national not-for-profit organisation, conducted a survey [15] of service users who identified gaps in support services, such as access to specialised ovarian cancer nursing care. The survey confirmed the desire for an ovarian cancer-specific, personalised case management service throughout individuals’ cancer experiences, echoing previous anecdotal feedback. In response to findings from the consumer survey and consultations with health professionals and other key partners, and informed by existing evidence, OCA established the Teal Support Program (TSP) in 2019, a nationwide telehealth case management service that connects individuals with specialised nurses, offering personalised support to address unmet needs and enhance person-centred care.

Using the RE-AIM framework [16], the TSP was evaluated independently to determine the program’s reach, effectiveness, adoption, implementation and maintenance. This paper presents findings on the program’s effectiveness, examining participants’ utilisation of TSP services, the impact of the program on their self-reported outcomes (PROMs), as well as their experiences and perceptions of the services. Findings related to other components of the framework will be reported separately.

## Methods

### Teal Support Program (TSP)

The aim of the program is to improve quality of life and reduce the burden for individuals living with ovarian cancer and their families by providing personalised supportive care through a case management approach led by specialist nurses delivered by telehealth. Individuals diagnosed with ovarian cancer can join the program through referrals from OCA services (e.g., OCA Facebook, Helpline), external health professionals or self-referral. The program is delivered by specialist oncology or gynaecology nurses with a minimum of five years’ experience caring for individuals with ovarian cancers. While participants continue to receive standard medical treatment and support, the program functions as an extension of their treating medical team, enhancing continuity and coordination of care.

Upon enrolment, each participant undergoes a comprehensive initial assessment conducted by an OCA specialist nurse. This assessment covers ovarian cancer and treatment-related symptoms, general and psychological health, and both immediate and long-term supportive care needs. Where relevant, the needs of carers, partners, and family members, as well as practical concerns, are also discussed. Following initial assessment, regular follow-ups are scheduled to provide ongoing support.

Support sessions involve nurse-led assessment, counselling and interventions, including: *Ongoing Support*, which encompasses assessment and intervention to address evolving needs throughout the care continuum; *Information Support*, including the provision of verbal guidance and complementary written and digital resources; *Care Coordination*, which includes communicating with the treating team to address identified needs; *Psychosocial Support*, comprising nurse-led counselling to address emotional, spiritual and social needs; and *Crisis Support*, which refers to non-scheduled sessions provided in response to urgent and acute needs. This level of support was introduced in 2021 with the addition of specialist ovarian cancer in-house psychologists and counsellors, to whom individuals with complex psychological, mental health needs or sexual health needs can be referred. Moreover, telehealth support sessions were complemented by access to OCA’s tailored ovarian cancer written information and support resources, as well as referrals to other relevant external resources and services as needed. OCA nurses aim to maintain direct communication with each participant’s oncology treatment team to ensure care is coordinated, integrated, and responsive to evolving needs. Regular multidisciplinary team meetings are conducted to discuss complex needs and provide further support where necessary. While all of the above supports were made available to each participant, not all participants required or accessed every support. Support was individualised and tailored to each participant's needs, stage in the cancer journey, personal preferences, and existing support resources.

### Study design

An embedded mixed methods design was used in this evaluation. Given the program’s purpose and nature, a prospective observational cohort study was conducted to assess its impact, guided by the RE-AIM framework [16]. This included an examination of participants’ PROMs over a 12-month period through multiple surveys. These quantitative data were complemented by conducting nested semi-structured interviews to further explore participants’ experiences with the program. The study was approved by the Queensland University of Technology (QUT) Human Research Ethics Committee (UHREC) (Approval number: 1900001068).

### Participants and setting

Between August 2020 and February 2022, all individuals over the age of 18 who had been diagnosed with ovarian cancer and enrolled to TSP were invited to participate in the evaluation by their OCA nurse. At enrolment to TSP, the nurse provided verbal information about the evaluation and, if interested, individuals were referred to the evaluation team. Individuals were provided with more detailed information, a Participant Information Sheet and any questions they had were addressed. Given the priorities of their cancer treatment and life circumstances, they were provided with as much time as needed to consider their involvement in the evaluation. Written consent was obtained to confirm their participation.

### Data collection

Participants were assigned a unique study ID for confidentiality and asked to complete surveys, either online or by paper questionnaire according to their preference, at the time of consent (baseline T_0_), and at 3 (T_1_), 6 (T_2_), and 12 (T_3_) months following their consent. The survey contained the full set of items from validated measures of ovarian cancer related symptom burden (Measure of Ovarian Symptoms and Treatment, MOST version 2 [17, 18]), psychological distress (General Health Questionnaire, GHQ-12 [19, 20]), quality of life (Functional Assessment of Cancer Therapy – Ovarian, FACT-O version 4 [21]), supportive care needs (Supportive Care Needs Survey short form, SCNS-SF34 [22]), and activation of self-management (Cancer Survivors Self-efficacy Scale, CS-SES [23]). Demographic and disease-related information of the participants were collected by the evaluation team upon receiving consents and at the T_0_ survey. In addition to the survey, usage of the TSP services by participants was collected as part of the OCA administrative records.

At consent, participants were additionally asked to indicate if they agreed to participating in an individual interview. Of those who agreed, thirty participants were then invited to share their experiences with the program and its perceived impact. They were purposively selected to represent the broader group of participants, ensuring diversity in age, duration since diagnosis, stage of cancer, treatment status, and residential location. These interviews were conducted by the author (W-HL) via telephone between six- and twelve-months post-consent, using a standardized Interview Guide, and were digitally recorded. The Interview Guide is provided in Supplementary File 1.

### Data analysis

Descriptive statistics were used to summarise the participants’ characteristics, TSP service uptake and PROMs. Spearman’s correlations (for continuous variables) or Mann–Whitney tests (for categorical variables) were used to explore the associations between PROMs and TSP service uptake and participants’ characteristics at baseline, such as age (< 60 years of age versus ≥ 60 years of age), residence area (metropolitan versus non-metropolitan), treatment status (whether or not undergoing systemic treatment) and duration of ovarian cancer diagnosis and TSP participation. The study was not designed as a comparative effectiveness study; general linear model repeated measures tests were employed to explore changes in participants’ self-reported outcome measures over a 12-month period adjusting for treatment status. Only participants who provided data at all time points were included in the test. All analyses were performed using SPSS for Windows (Version 30.0). An alpha level of p ≤ 0.05 was considered statistically significant.

For interview data, the audio recordings of the interviews were transcribed verbatim by a professional transcriber. The length of the interviews varied from 15 to 60 min. Exploratory thematic analysis was conducted independently by two researchers (W-HL, VO) using an inductive approach. This involved reading the transcripts closely, generating initial codes, categorising similar meaning units, and grouping into broader themes, reviewing and defining themes, following the steps outlined by Braun and Clarke [24]. Discrepancies were discussed until mutual agreement was reached. The third researcher (PY) checked the themes identified.

## Results

### Characteristics of participants

**Fig. 1:**
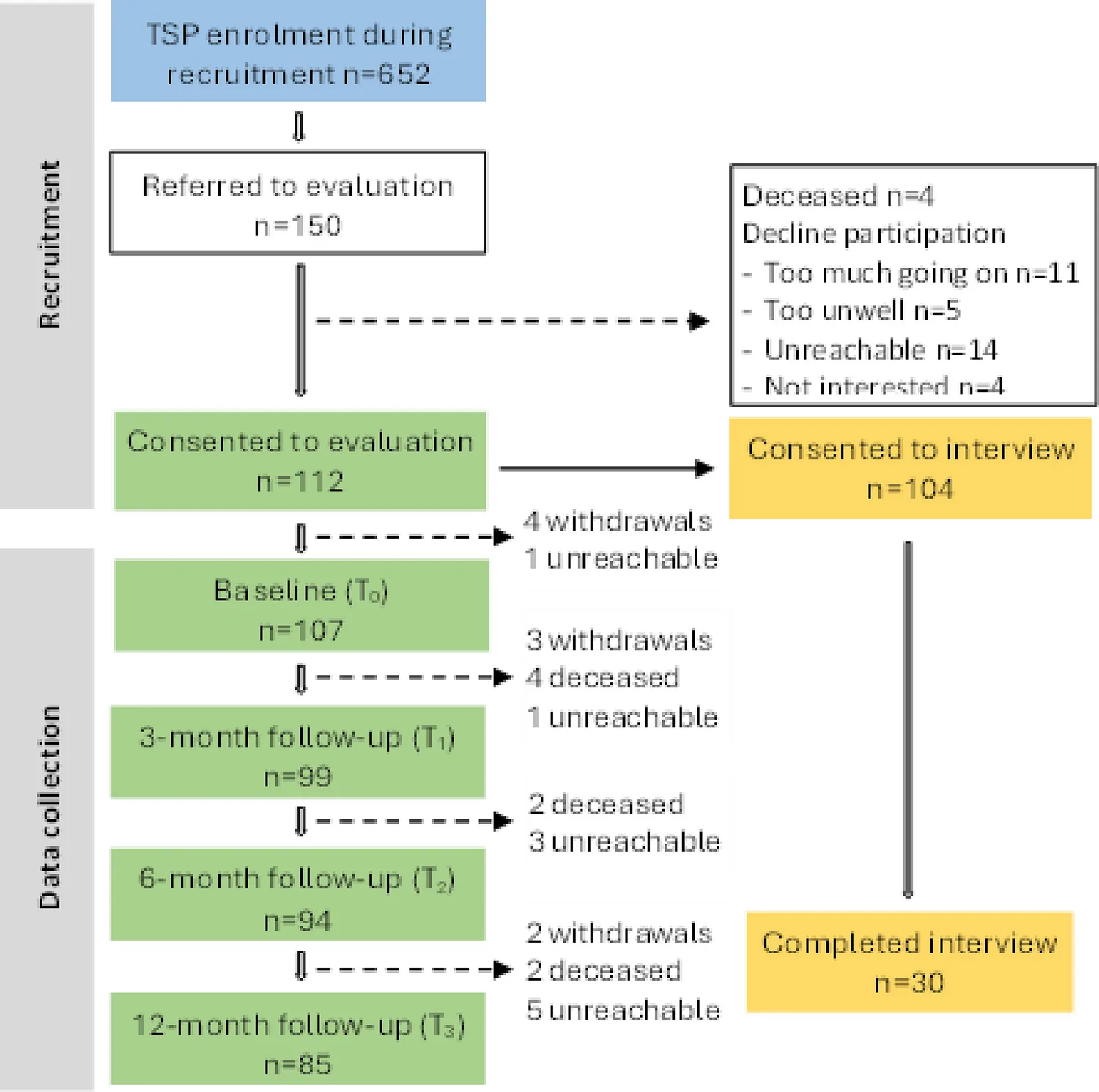
Recruitment and data collection flowchart of the evaluation study

A total of 652 individuals enrolled in the TSP program during the recruitment period. Of these, 150 were referred for evaluation, and 112 (17%) consented to participate in the evaluation. Among those who consented, 104 also agreed to be interviewed. The flowchart of the recruitment and data collection procedures is presented in Fig. 1. Seven out of nine withdrawals were due to a rapid deterioration in health status, while the other two expressed they did not want to participate in surveys.

The demographic and clinical characteristics of the participants at baseline are summarised in Table 1. The median age of the participants was 61.0 years (range 28–85) and 44% (49/111) were under 60 years of age. The median time since ovarian cancer diagnosis was 13 months (range 1–124). Most participants (93/105) had received surgical treatment, and 62% (69/111) were undergoing systemic treatment (e.g., chemotherapy, hormone, targeted, or immunotherapy). The median length of TSP participation was five months (range 1–23) and 63% (70/111) had participated in TSP for over three months at consent.

**Table 1:** Baseline demographic and clinical characteristics of participants (*n*=112)

Characteristics		n (%)
Age groups (years) (n=111)	<40	4 (4)
	40-49	13 (12)
	50-59	32 (29)
	60-69	35 (31)
	≥70	27 (24)
Area of residence (n=112)	Major cities	75 (67)
	Inner regional	27 (24)
	Outer regional/remote	10 (9)
Marital status (n=106)	Married/de facto	79 (75)
	Widowed/divorced/separated/never married	27 (25)
Education level (n=106)	Primary school	1 (1)
	Secondary school/TAFE college	51 (48)
	University degree or higher	54 (51)
Employment status (n=106)	Working full/part-time/casual	31 (29)
	Retired/home duties/unemployed	52 (49)
	Sick/on leave/disability	23 (22)
Annual individual gross income* (n=105)	< AUD60,000	53 (51)
	≥ AUD60,000	32 (30)
	Do not wish to disclose	20 (19)
Being a carer to dependents (n=103)	Dependent child	14 (14)
	Aged spouse/person with a disability	10 (10)
	No dependents	79 (77)
Time since ovarian cancer diagnosis at consent (n=111)	≤6 months	27 (24)
	7-12 months	28 (25)
	13-24 months	28 (25)
	25-36 months	6 (5)
	37-48 months	11 (10)
	> 48 months	10 (10)
Time since TSP enrolment at consent (n=111)	≤1 month	12 (11)
	2-3 months	29 (26)
	4-6 months	39 (35)
	7-12 months	27 (24)
	>12 months	4 (4)
Stage of cancer at diagnosis (n=111)	I/II	21 (19)
	III/IV	83 (75)
	Do not know	7 (6)
Phase in treatment at consent (n=112)	Initial treatment	39 (35)
	Following initial treatment	27 (24)
	First recurrence	19 (17)
	Second/multiple recurrence	10 (9)
	Platinum resistant	5 (4)
	In remission (no treatment)	11 (10)
	End stage	1 (1)
Undergoing systemic treatments (n=111)	Yes	69 (62)
	No	42 (38)

*2021-22 Australian median total personal income was AUD55,062 according to https://www.abs.gov.au/statistics/labour/earnings-and-working-conditions/personal-income-australia/latest-release

### Baseline PROMs and association with participants’ characteristics

As a whole group, fatigue (69/106, 65%), anxiety (59/106, 56%), and trouble sleeping (53/106, 50%) were the most common moderate-to-severe symptoms. Less than half of participants reported good or very good physical (46/105, 44%), emotional (42/105, 40%), or overall well-being (48/105, 46%). Participants reported a mean of 3.7 (SD = 3.4) psychological distress items (maximum possible score = 12). Overall, 18% (19/106) reported no psychological distress, while 43% (46/106) reported high psychological distress (mean score ≥ 4). Emotional wellbeing was the lowest-scoring FACT-O domain. The most common moderate-to-high unmet supportive care needs were uncertainty about the future (58/107, 54%), fears of cancer spreading (51/106, 48%), anxiety (44/106, 42%), feeling in control of their situation (44/106, 42%), and concern that treatment outcomes were beyond their control (44/105, 42%). About 44% (47/107) of the participants had some concerns about sexuality. Few participants reported no unmet needs, with 26% (28/106) reporting no health system/information needs; 23% (25/107) no physical or daily living needs, and 9% (10/107) no psychological needs. Most participants were confident or very confident in accessing information (79/104, 76%), support services (80/104, 77%), contacting a doctor about problems (74/105, 71%), and obtaining help when needed (78/105, 74%); however, around one-quarter reported low confidence in dealing with problems (29/105, 28%), fatigue (28/104, 27%) and distress (24/104, 23%). The mean scores of the various PROMs at baseline are summarised in Table 2.

**Table 2:** Changes in patient-reported outcomes over 12-month period

PROMs		Baseline		3-month		6-month		12-month		General Linear Model
		n	Mean (SD)	n	Mean (SD)	n	Mean (SD)	n	Mean (SD)	
**Symptoms & wellbeing**^a^	Abdominal (MOST-Abdo)	106	27.0 (25.8)	98	22.6 (24.3)	94	24.6 (26.3)	84	24.6 (24.7)	n=77* p*=0.51 F(3, 225)=0.77
	Disease/treatment-related (MOST-DorT)	106	21.4 (15.5)	99	19.4 (14.0)	94	21.0 (17.1)	84	20.2 (16.5)	n=77* p*=0.45 F(3, 225)=0.88
	Chemotherapy-related (MOST-Chemo)	106	21.7 (17.0)	99	16.6 (14.5)	94	15.6 (14.4)	84	15.9 (15.5)	n=77* p*=0.21 F(3, 225)=1.51
	Psychological (MOST-Psych)	106	35.7 (24.7)	99	30.6 (25.7)	94	32.3 (24.1)	84	29.6 (25.6)	n=77* p*=0.47 F(3, 225)=0.84
	Wellbeing (MOST-Wellbeing)	105	57.7 (18.2)	98	63.1 (18.8)	94	60.6 (19.1)	85	63.8 (18.3)	n=75p =0.047 F(3, 219)=2.70*
**Psychological **^b^	Level of distress	106	3.7 (3.4)	99	3.3 (3.7)	94	3.3 (3.4)	84	3.4 (3.7)	n=77* p*=0.52 F(3, 225)=0.76
**Quality of life **^c^	Physical wellbeing (PWB)	106	19.9 (5.2)	99	20.5 (5.3)	94	20.4 (5.4)	84	19.9 (6.2)	n=77* p*=0.23 F(3, 225)=1.45
	Social/Family wellbeing (SWB)	106	20.6 (5.6)	98	20.2 (5.5)	94	20.6 (5.6)	83	21.3 (5.3)	n=77* p*=0.26 F(3, 225)=1.35
	Emotional wellbeing (EWB)	105	12.6 (4.0)	98	12.9 (4.2)	94	12.8 (4.3)	83	12.4 (4.8)	n=75* p*=0.51 F(3, 219)=0.77
	Functional wellbeing (FWB)	106	16.7 (5.1)	99	17.1 (5.3)	94	16.6 (5.8)	84	17.4 (5.8)	n=77* p*=0.89 F(3, 225)=0.20
	Ovarian cancer specific concerns (OCS)	106	30.5 (5.5)	99	31.8 (5.8)	94	31.0 (6.5)	84	31.2 (6.6)	n=77* p*=0.35 F(3, 225)=1.09
	FACT-O Total score	105	100.1 (19.3)	97	102.5 (20.5)	94	101.4 (21.7)	83	102.4 (22.8)	n=75* p*=0.13 F(3, 219)=1.94
**Supportive care needs** ^d^	Physical & daily living	107	2.3 (1.7)	98	2.2 (1.9)	94	2.2 (1.9)	85	2.2 (1.8)	n=78* p*=0.90 F(3, 228)=0.20
	Psychological	107	6.3 (3.4)	98	5.8 (3.7)	94	5.9 (3.6)	85	5.8 (3.8)	n=78* p*=0.63 F(3, 228)=0.58
	Sexuality	107	1.0 (1.3)	98	0.9 (1.2)	94	0.9 (1.3)	85	0.8 (1.2)	n=78* p*=0.41 F(3, 228)=0.97
	Patient care & support	107	1.4 (1.7)	98	1.2 (1.7)	94	1.6 (2.0)	85	1.4 (1.8)	n=78* p*=0.09 F(3, 228)=2.22
	Health system & information	106	3.7 (3.7)	98	2.9 (3.4)	94	3.7 (3.9)	85	3.3 (3.8)	n=78 p=0.027 F(3, 228)=3.11**
**Self-efficacy **^e^	Total score	105	6.8 (1.7)	99	6.9 (1.8)	94	6.7 (2.0)	84	6.7 (2.0)	n=77* p*=0.38 F(3, 225)=1.02

Whether or not receiving active systemic treatment at baseline was adjusted as covariate in the model.

*Partial Eta Squared = 0.04; **Partial Eta Squared = 0.04.

Score range: ^a^ MOST 0-100; ^b^GHQ 0-12; ^c^PWB 0-28, SWB 0-28, EWB 0-24, FWB 0-28, OCS 0-44, FACT-O total 0-152; ^d^ Number of items reporting some need in each domain: physical and daily living 0-5, psychological 0-10, sexuality 0-3, patient care and support 0-5, health system and information 0-11; ^e^ 1-10. SD = Standard deviation

Higher scores represent ^a^ worse symptoms and better wellbeing; ^b^ higher level of distress; ^c^ better quality of life; ^d^ more unmet needs; ^e^ higher self-efficacy.

The correlations between baseline PROMs are summarised in Supplementary File 2. Except for sexuality-related concerns, higher levels of needs across all domains were significantly associated with greater ovarian cancer symptom burden and distress, and with lower wellbeing and self-efficacy. At consent, participants receiving systemic treatments reported significantly higher psychological distress (*p* < 0.01), greater disease- or treatment-related symptoms (*p* = 0.02), poorer physical well-being (*p* < 0.01), higher needs in sexuality (*p* = 0.01), and lower confidence in self-management (*p* < 0.05) than those not receiving active systemic treatment.

**Fig. 2:**
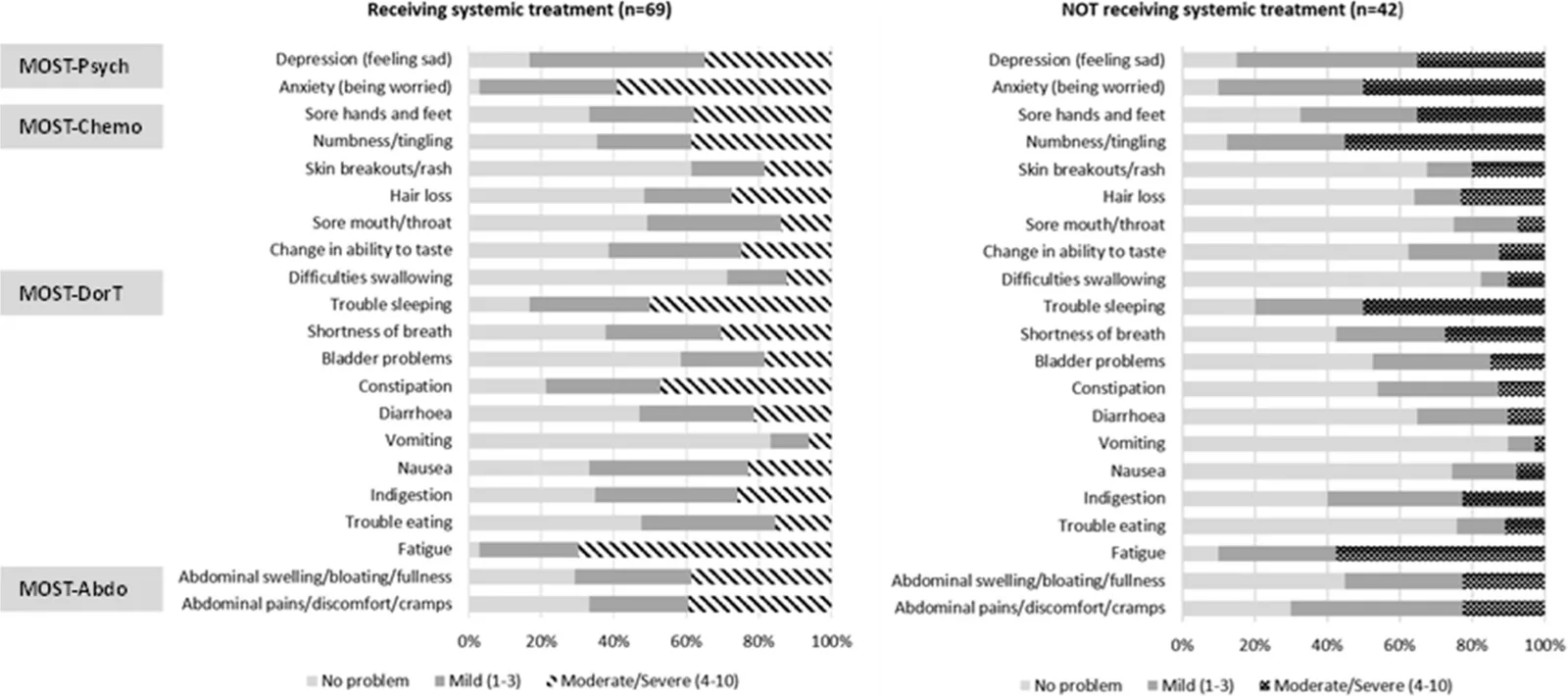
The prevalences of ovarian cancer symptoms by treatment status at baseline. Note: Each item may have different numbers of missing data

Figure 2 shows the prevalences of ovarian cancer symptoms by treatment status at baseline. Approximately 69% (34/49) of participants aged < 60 years were receiving systemic treatment, compared with 57% (35/62) of those aged 60 and above. Younger participants reported more severe disease- or treatment-related (*p* = 0.03) and abdominal (*p* = 0.03) symptoms, poorer quality of life in physical (*p* < 0.01), social (*p* = 0.03), and overall well-being (*p* = 0.03), and greater sexuality (*p* < 0.01) and information (*p* < 0.01) needs than older participants. Compared with participants living in major cities, those in non-metropolitan areas reported significantly better quality of life in social, functional, ovarian cancer specific, and overall wellbeing (all *p* < 0.01), lower distress (*p* < 0.01), less severe disease- or treatment-related symptoms (*p* = 0.04), and lower needs in sexuality (*p* < 0.01), information (*p* < 0.01), care and support (*p* < 0.05), and psychological (*p* = 0.03) domains. Social wellbeing was negatively associated with time since ovarian cancer diagnosis (*p* = 0.01). No other baseline PROMs differed by time since ovarian cancer diagnosis or TSP enrolment.

### TSP services utilisation

A total of 2781 episodes of TSP services were delivered to participants from the time of enrolment in the TSP until the end of the 12-month follow-up period in this evaluation. The utilisation details of each TSP service type are presented in Table 3. Most participants (80%) received information support and one-third received specialised psychosocial support. The total number of TSP service episodes received by each participant did not differ by age (*p* = 0.99), residence location (*p* = 0.12), or TSP enrolment duration (*p* = 0.60). Participants receiving systemic treatment at consent received significantly more care coordination episodes than those not receiving treatment (*p* < 0.01).

**Table 3:** Utilisation of TSP services by participants (*n*=102*)

Purpose of TSP service	No. of episodes	No. of participants (%)	No. of episodes per participantMedian (range)
Ongoing support (scheduled)	1661	101 (99)	12 (1-83)
Information support	542	82 (80)	6 (1-27)
Care coordination	114	40 (39)	3 (1-11)
Psychosocial support	256	34 (33)	6 (1-24)
Crisis support (Non-scheduled)	90	32 (31)	1 (1-17)
Other purposes	118	39 (38)	1 (1-10)
**Total TSP services**	**2781**	**102 (100)**	**23 (1-111)**

From each participant’s enrolment in the TSP until the end of the 12-month follow-up period.

* TSP service records were available for 102 participants, excluding initial assessment.

Supplementary File 3 presents the associations between participants’ utilisation of TSP services and their baseline PROMs. Participants with greater disease- or treatment-related (*p* = 0.02) and psychological (*p* < 0.01) symptoms, higher distress (*p* = 0.02), poorer quality of life in physical (*p* = 0.04), social (*p* = 0.02), functional (*p* = 0.02), ovarian cancer specific (*p* < 0.01) and overall wellbeing (*p* < 0.01), and lower self-efficacy (*p* < 0.01) at baseline received more TSP services overall. Information support was associated with poorer overall wellbeing (*p* = 0.03). Participants with greater disease- or treatment-related symptoms (*p* < 0.05), higher distress (*p* < 0.01), poorer quality of life in physical (*p* = 0.04) and ovarian cancer specific (*p* = 0.04) wellbeing received more care coordination services.

### Changes in PROMs over time

After adjusting for participants’ baseline treatment status, statistically significant but small improvements were observed over time during TSP participation in information needs (*p* = 0.03) and overall wellbeing (as measured by MOST-Wellbeing; *p* < 0.05), as shown in Table 2.

### Participants’ experience and perceptions of TSP services

During the interviews, participants described various ways in which TSP services supported them and the extent to which these services positively impacted their quality of life and care experiences. The services were consistently recognised for their value in addressing individuals’ concerns and meeting their supportive care needs over time. Key themes that emerged from the data included: (a) being seen and heard as a whole person, (b) receiving trusted and personalised information and advice, (c) experiencing meaningful and personalised emotional support, and (d) accessing services that are flexible and timely.

### Being seen and heard as a whole person

Participants frequently shared examples of how their interactions with TSP services addressed a broad spectrum of wellbeing needs, ranging from symptom management and emotional support to practical concerns and attention to personal circumstances. The nurses’ approach fostered a sense of personal connection, trust, and relational continuity, making participants feel seen and heard as whole individuals, rather than simply as patients.

*It’s sort of a whole approach sort of thing. She’ll say you know “how is the family and are you cutting down on your stress? Just look after yourself remember nutrition, remember the exercising and remember to let the brain have a rest” and all that sort of thing.* (ID008, 60–64 years old, diagnosed 5–10 years at interview, undergoing treatment for first recurrence, living in regional area.)

*You have the feeling that they (the OCA nurse) have unlimited time to give you which is very rare with all these other health professionals. Where mostly you are feeling time pressure every time you talk to them. And also, they’re more like just a friend as well as a nurse so that you can literally talk about anything. And it’s surprising the number of things that you feel you can’t talk about with the health professionals. Like your oncologist or whatever. Or family or friends. So I feel like the Teal program has been essential in a way to, for so many reasons. To keep my mood buoyant.* (ID082, 55–59 years old, diagnosed 1–2 years at interview, completed initial treatment, living in metropolitan area.)

One participant (ID016) provided extensive examples of how the OCA nurse supported her across multiple domains. This included assistance in understanding genetic test results, referrals to relevant specialists for specific issues, guidance on managing chemotherapy side effects (e.g., hair loss and mouth care), and facilitating peer connections with other individuals diagnosed with ovarian cancer. The nurse also provided information on emerging treatment options, offered emotional support during periods of isolation and loneliness, and demonstrated concern for her family’s wellbeing, ensuring that she received both familial and social support. As the participant stated:

*So there’s definitely not a personal connection there with the gyn/onc team in xxx (the hospital). They are very professional and they’re very friendly and they ring and do their telehealth appointment but they’re just, they’re basically going “What’s your CA125 level? Oh it’s normal good, good, good, good luck bye”. They’re not going to be there to help me on a personal or day to day level. So as far as xxx (the OCA nurse) was the, xxx is my only point of medical care specialist for ovarian cancer who supports me in many spectrums. Social, emotional, practical, medical, clinical. She’s the only person, if it wasn’t for her, I don't know I would have been just more lost. More lost and more alone and more confused and more struggling.* (ID016, 45–49 years old, diagnosed 1–2 years at interview, completed initial treatment, received care at multiple hospitals, living in regional area.)

### Receiving trusted and personalised information and advice

Participants consistently described the OCA nurses as trusted source of information. This support extended beyond the provision of educational materials. Nurses proactively sourced, clarified, and verified information from other health professionals on behalf of them, thereby enhancing access to timely and tailored guidance. The information provided was not only clinically relevant, addressing symptoms such as post-treatment menopause and skin issues during or after treatment, as well as treatments, medications, tests and clinical trial opportunities, but also helped raise awareness of available resources and support services. As one participant described, “*She (the OCA nurse) opened up my landscape of things I didn’t know about*” (ID016). Importantly, participants reported that the information helped them make sense of their symptoms and emotional experiences, reducing anxiety and supporting their mental wellbeing.

*The dedicated support nurses are worth their weight in gold because they are able to talk about medications, they are able to talk about things that are in the pipeline. They’re able to talk about research. And that was very, very important to me at the time.* (ID004, 55–59 years old, diagnosed 1–2 years at interview, undergoing initial treatment, living in metropolitan area.)

*I found that a lot of the information that I was given in hospital was just general cancer, it wasn’t specific for ovarian. So I was looking for more specific information about my cancer. There was a lot of stuff out there for cancer in general, a lot of stuff about breast cancer but not a lot about ovarian cancer. So the Teal program made it that I could get that information.* (ID063, 55–59 years old, diagnosed 1–2 years at interview, completed initial treatment, living in regional area.)

*Because to get the [palliative] services you need a referral obviously, so I’ll sit and discuss what he (the General Practitioner) knows about them. Because it’s a real learning curve finding out what’s available. What’s, how much it costs, whether they come to xxx (name of residential area) or not, all of these things. And that’s where the Ovarian Cancer Australia nurse has been extremely helpful in that she’s been, she’s done some of that and advised with some of that. So that when I go into my GP, I will actually have a little bit of knowledge, and I’ll be a little bit pre-prepared.* (ID090, 70–74 years old, diagnosed 5–10 years at interview, undergoing palliative chemotherapy, living in metropolitan area.)

### Experiencing meaningful and personalised emotional support

Participants acknowledged that the OCA nurses provided meaningful support in managing issues that contributed to emotional distress. This support was delivered through reassurance and validation of their feelings, which helped alleviate their worries and depression. One woman noted that the nurse ‘*helped me reframe my mental state because it wasn’t good at that time*’. (ID016). The TSP services were described as flexible and responsive to individual circumstances, with nurses available at critical moments to offer support when it was most needed.

*… just to have someone there that understood exactly what you were talking about, someone that obviously had dealt with a lot of women going through the same thing. So, you know just to be reassured that you’re not the only one out there that’s feeling like that I think helped a lot. … She (the OCA nurse) was soothing.* (ID048, 50–54 years old, diagnosed 1–2 years at interview, completed initial treatment, living in regional area.)

*[The biggest benefit was] that I had someone to talk with particularly at the beginning you know. At the beginning when you’re diagnosed the first month, two months they [are a] rollercoaster. You don't know where you are and how you are and particularly with chemo, they (the OCA nurse) helped me. So they were giving me this kind of support, and throughout the chemo as well. Which was really important. Because sometimes you panic, and you think you don't know what’s normal and what’s not normal. … And so it’s good to have that support.* (ID029, 45–49 years old, diagnosed 1–2 years at interview, completed initial treatment, living in metropolitan area.)

*… it’s like childbirth I suppose you know. When people tell you this is what it’s like, I think it’s different to experience it. I think that was the most horrible moment especially the first few days after chemo when the chemo sets in. It was that specific moment to me, was the hardest when I think about it. … So that was a very difficult time for me, and the Teal Support people sort of said it’s alright, it’s quite normal, you’ll get yourself into a pattern. The little incidents that happen but they were there.* (ID054, 55–59 years old, diagnosed <1 year at interview, completed initial treatment, living in metropolitan area.)

### Accessing services that are flexible and timely

Most participants noted that there was no consistent or ideal time for receiving support, as their needs varied depending on their stage and personal circumstances. Some shared that they could reach out to the nurse whenever needed via telephone support. The frequency of contact was also adaptable, tailored to each individual's situation. This flexible approach made participants felt someone was always there for them.

*When I started having this fluid problem (after surgery) I was actually able to send her the video of the fluid moving around. Because we knew how to use technology now so it’s much better than having to go to a place that was in lockdown because of Covid. And then yeah just … you’re not spending all that time travelling and you know you’re not wearing yourself out. … For me to be here at nine o’clock I will have to get up at five, leave at five thirty to get here for nine o’clock.* (ID008, 60–64 years old, diagnosed 5–10 years at interview, undergoing treatment for first recurrence, living in regional area.)

*You can call them any time. When I was really low, I was speaking with them weekly. Now we have monthly check-ins but basically, they’re there for you whenever you really need them. So you can just make a call, and someone will call you back almost immediately so yeah.* (ID004, 55–59 years old, diagnosed 1–2 years at interview, undergoing initial treatment, living in metropolitan area.)

A few participants expressed a preference for face-to-face support, noting that telephone contact alone was not always sufficient. As one participant explained, ‘*I just feel I just can’t do it over the phone and in a short time. I probably have too much (stress), just doesn’t work for me.*’ (ID057). Others shared that while they did not rely solely on the TSP support, they valued having it as a second source of support.

*I felt having that second because it (the TSP) wasn’t as if it was my only support. I felt yeah by the phone was fine. If I’d had to go and try and make an appointment and see somebody face-to-face it probably would have been just another thing to organise. And I felt really connected on the phone that I could get the information I needed. I would have been happy to do that rather than go and see someone.* (ID056, 40–44 years old, diagnosed <1 year at interview, undergoing initial treatment, living in regional area.)

## Discussion

This study evaluated the impact of a nationwide telehealth case management service for individuals with ovarian cancer, focusing on their self-reported outcomes as well as their experiences and perceptions of the service. Quantitative findings from this observational study demonstrated statistically significant, albeit small, improvements in overall wellbeing and satisfaction with information received over the 12-month study period. Statistically significant changes were not observed over time in ovarian cancer specific symptoms, psychological distress, self-efficacy and unmet needs in other areas that may be impacted by the nature and typical progression of ovarian cancer. Importantly, the qualitative findings consistently revealed that participants’ experiences with the program were consistently positive. Taken overall, participants’ experience of the program reflected that it effectively addressed their key concerns related to symptom burden, emotional and mental health challenges, and unmet needs.

The patient-reported outcomes from our quantitative data reinforce a growing body of evidence indicating that individuals with ovarian cancer experience complex and multifaced supportive care needs. Notably, our participants were younger than the broader Australian ovarian cancer population [1], with our findings revealing that participants under 60 reported significantly greater disease- and treatment-related symptom burden, higher needs around sexuality and information, and poorer quality of life compared to those aged 60 and over. Such differences likely reflect the distinct psychological and social challenges faced by younger individuals with ovarian cancer, who are often at stages of family caregiving and career advancement [25]. They may also be influenced by the higher proportion of younger participants undergoing systemic treatment compared to those aged 60 and over in this study. Our findings highlight the importance of incorporating regular supportive care needs assessment to inform individualised, person-centred care that addresses the diverse needs of individuals based on their life circumstances, age, and treatment status; in alignment with the Optimal Care Pathway for Ovarian Cancer [26].

Of note, rates of anxiety and depression among our cohort were markedly higher than those reported in previous studies [6]. Participants also experienced more severe fatigue, anxiety, and sleep disturbances than those in a large international study of recurrent ovarian cancer using the same assessment tool [18]. These findings suggest that participants accessing the tailored TSP services represent a subgroup with elevated supportive care needs and a greater risk of poorer health outcomes compared to the broader ovarian cancer population. This highlights the value of providing additional services beyond standard care for a cancer population known to commonly have higher levels of needs.

Consistent with other research on unmet needs of individuals with ovarian cancer [25, 27], our results also revealed significant associations between participants’ needs across multiple domains and their symptom burden, psychological distress and wellbeing. Both quantitative and qualitative data showed that TSP services were responsive and demonstrated some effectiveness in addressing these needs. For instance, nearly three-quarters of participants reported information needs, and service records confirmed that most of them received information support. Participants with poorer overall wellbeing received more frequent assistance, reflecting a responsiveness that is necessary for effective and efficient delivery of supportive care services. Interview data further highlighted the pivotal role of these specialist nurses as a trusted source of information and advice, most likely contributing to the significant improvement in satisfaction with information received over time. This finding aligns with prior research showing that information provision is associated with reduced unmet supportive care needs among individuals with ovarian cancer [28]. Psychological distress was another major concern, with 43% of participants reporting high levels. Interview data consistently highlighted the value participants placed on the emotional and holistic supportive care provided by nurses. Additionally, 33% accessed OCA’s specialised psychosocial support services, indicating that the program effectively identified and responded to more complex needs. This inclusion of multidisciplinary services reflects a comprehensive, person-centred model of care, consistent with internationally recognised supportive care in cancer goals [29] and the principles outlined in the Integrated Cancer Care Framework [12]. These findings demonstrate the critical role of TSP services in reaching a subgroup of individuals with higher needs and delivering personalised care to those with more complex and diverse needs. Overall, this observational study shows consistent findings across quantitative and qualitative data, demonstrating the value of the TSP for those who used it.

While a small number of participants indicated a preference for face-to-face contact, the overall findings suggest that telehealth is an acceptable and effective model of supportive care for individuals affected by ovarian cancer. This finding aligns with a growing body of literature that demonstrates the value of telehealth interventions for individuals with cancer and other chronic conditions. Telephone or telehealth follow-up led by nurses after cancer treatment has been shown to be convenient, cost- and time-saving, and effective in delivering consistent care across both urban and rural areas. It is also feasible for assessing a large number of patients, well accepted by patients, and has demonstrated benefits in reducing symptom burden and emotional distress, while improving quality of life and self-care among individuals with cancer [30–32]. In the context of ovarian cancer, evidence for effective follow-up strategies remains limited [33]. One UK study [34] found that nurse-led individualised follow-up, primarily conducted by telephone, enhanced health-related quality of life and patient satisfaction among individuals with ovarian cancer, compared to standard medical follow-up. Similarly, another UK study [35] reported that nurse-led telephone follow-up was not only acceptable and convenient but also led to notable improvements in individuals’ emotional wellbeing. In Australia, a trial [36] is currently underway to evaluate nurse-led telephone follow-up focused on identifying and managing psychosocial symptoms, late treatment side effects, and signs of recurrence. All of the services described in the studies above were limited to hospital settings and accessible only to individuals receiving care at participating sites. The TSP was one of the first freely accessible, nationwide nurse-led telehealth programs offering supportive care, with goals and delivery methods comparable to previous studies, but designed for broader reach to ensure equitable access at a national scale. Demographic and clinical data from our study confirm that participants represented a diverse sample, spanning all stages of the cancer trajectory, age groups, and geographical locations. Both quantitative and qualitative findings suggest that the TSP model has strong potential to achieve its intended goals by delivering services that are not only accessible but also highly responsive to the unique and evolving needs of individuals throughout their cancer journey. Beyond the delivery of the TSP evaluated in this study, the program continues to evolve and improve. It is now embedded within the national Australian Cancer Nursing and Navigation Program to support the goals of the Australian Cancer Plan [37]. Expanding data capture to assess service reach for diverse and priority populations, along with integrating digital PROMs for clinical use, exemplifies opportunities to further evaluation of the TSP in future research.

One of the key strengths of this study was using mixed methods design. Individual interviews with a subset of participants added depth to the quantitative findings, providing rich data illustrating the lived experiences of individuals with ovarian cancer. The qualitative data provided critical insights into how participants were supported, what their concerns were, and the extent to which those concerns were addressed. However, this evaluation study also had several limitations. First, due to its observational nature and the absence of a controlled experimental design, caution is needed when interpreting changes in PROMs as direct effects of the program. This limitation is somewhat mitigated by the use of multiple sources of quantitative and qualitative data which provided valuable context, revealing how and to what extend the service addressed unmet needs. Additionally, most participants in this study sample had already been using the TSP prior to consenting to the evaluation, meaning baseline data were not collected at the point of program enrolment. Moreover, of the 652 individuals enrolled in the TSP program during the evaluation period, only 150 were referred for participation in the evaluation. Feedback from the nurses indicate that non-referral was often due to individuals being too unwell or preferring not to be referred, which is understandable given the high disease-related burden in this population. We acknowledge the potential for response bias, as those who participated may have been more motivated to contribute to the program’s evaluation. However, TSP service utilisation did not differ by age, residential location, or length of program participation. Instead, higher service utilisation was linked to more severe disease- or treatment-related symptoms, greater psychological distress, and lower levels of quality of life and self-efficacy. This suggests that the program was effectively being tailored to individual needs, prioritising supportive care for individuals at greater risk of poor health outcomes or with significant unmet needs. It is important to note that the broad quantitative categorisation of service activity records limits our ability to capture the complexity of the support provided. Due to the dynamic and responsive nature of supportive care provision, nurses often addressed multiple concerns within a single contact or across the course of care, making it difficult to attribute specific outcomes to individual support categories. For instance, activities classified as care coordination or information provision frequently encompassed psychosocial elements. Moreover, information provision played a critical role in addressing not only clinical and practical concerns but also psychosocial needs. This highlights the challenge of linking specific support activities to distinct outcomes.

## Conclusions

This evaluation provides important evidence supporting the effectiveness of a nationwide, nurse-led, telehealth program in addressing the complex supportive care needs of individuals with ovarian cancer. The observed improvements in patient-reported outcomes, along with the positive experiences shared by participants, suggest that the program has made a meaningful contribution to enhancing quality of life across individuals’ ovarian cancer experiences and has great potential for this population. These findings highlight the need for continued investment in models of supportive cancer care that prioritise emotional wellbeing, symptom management, and overall quality of life. The program offers a strong example of person-centred approach that could inform future national initiatives broadly across cancer care.

## Supplementary Information

Below is the link to the electronic supplementary material.

Supplementary file1 (DOCX 37 KB)

## Data Availability

Data described in the manuscript will be made available upon reasonable request.

## References

[1] Australian Institute of Health and Welfare (2024). Cancer data in Australia. Canberra: Australian Institute of Health and Welfare. Available from: https://www.aihw.gov.au/reports/cancer/cancer-data-in-australia. Accessed 30 June 2025.

[2] Hennessy BT, Coleman RL, Markman M (2009) Ovarian cancer. Lancet 374:1371–1382 10.1016/S0140-6736(09)61338-6 19793610

[3] Lheureux S, Gourley C, Vergote I, Oza AM (2019) Epithelial ovarian cancer. Lancet 393:1240–1253 10.1016/S0140-6736(18)32552-2 30910306

[4] Logue CA, Pugh J, Jayson G (2020) Psychosexual morbidity in women with ovarian cancer. Int J Gynecol Cancer 30(12):1983–1989 10.1136/ijgc-2020-002001 PMC7788483 33115791

[5] Ozga M, Aghajanian C, Myers-Virtue S et al (2015) A systematic review of ovarian cancer and fear of recurrence. Palliat Support Care 13(6):1771–1780 10.1017/S1478951515000127 PMC4995592 25728373

[6] Watts S, Prescott P, Mason J et al (2015) Depression and anxiety in ovarian cancer: a systematic review and meta-analysis of prevalence rates. BMJ Open 5:e007618 10.1136/bmjopen-2015-007618 PMC4679843 26621509

[7] Ahmed-Lecheheb D, Joly F (2016) Ovarian cancer survivors’ quality of life: a systematic review. J Cancer Surviv 10:789–801 10.1007/s11764-016-0525-8 26884372

[8] Lee YC, King MT, O’Connell RL et al (2022) Symptom burden and quality of life with chemotherapy for recurrent ovarian cancer: the Gynecologic Cancer InterGroup-Symptom Benefit Study. Int J Gynecol Cancer 32(6):761–768 10.1136/ijgc-2021-003142 PMC9185817 35086926

[9] Webber K, Carolus E, Mileshkin L et al (2019) OVQUEST – life after the diagnosis and treatment of ovarian cancer - an international survey of symptoms and concerns in ovarian cancer survivors. Gynecol Oncol 155(1):126–134 10.1016/j.ygyno.2019.08.009 31416612

[10] Beesley VL, Ross TL, King MT et al (2022) Evaluating patient-reported symptoms and late adverse effects following completion of first-line chemotherapy for ovarian cancer using the MOST (measure of ovarian symptoms and treatment concerns). Gynecol Oncol 164(2):437–445 10.1016/j.ygyno.2021.12.006 34955238

[11] Nho J-H, Kim SR, Nam J-H (2017) Symptom clustering and quality of life in patients with ovarian cancer undergoing chemotherapy. Eur J Oncol Nurs 30(10):8–14 10.1016/j.ejon.2017.07.007 29031318

[12] Krishnasamy M, Hyatt A, Chung H et al (2023) Refocusing cancer supportive care: a framework for integrated cancer care. Support Care Cancer 31(1):14 10.1007/s00520-022-07501-9 PMC9747818 36513841

[13] Clinical Oncology Society of Australia Model of Survivorship Care Working Group (2016) Model of survivorship care: Critical components of cancer survivorship care in Australia Position Statement. Clinical Oncology Society of Australia.

[14] Lynex C, Meehan D, Whittaker K et al (2025) The need for improved integration of psychosocial and supportive care in cancer: a qualitative study of Australian patient perspectives. Support Care Cancer 33(6):516 10.1007/s00520-025-09593-5 PMC12125069 40445269

[15] Pasvanis M, Hegarty S, Russell H et al (2023) Exploring the experiences and priorities of women with a diagnosis of ovarian cancer. Support Care Cancer 31(7):432 10.1007/s00520-023-07903-3 PMC10313536 37389743

[16] Glasgow RE, Vogt TM, Boles SM (1999) Evaluating the public health impact of health promotion interventions: the RE-AIM framework. Am J Public Health 89(9):1322–1327 10.2105/ajph.89.9.1322 PMC1508772 10474547

[17] King MT, Stockler MR, Butow P et al (2014) Development of the Measure of Ovarian Symptoms and Treatment Concerns: aiming for optimal measurement of patient-reported symptom benefit with chemotherapy for symptomatic ovarian cancer. Int J Gynecol Cancer 24:865–873 10.1097/IGC.0000000000000167 24844220

[18] King MT, Stockler MR, O’Connell RL ... for the GCIG Symptom Benefit group (2018) Measuring what matters MOST: validation of the Measure of Ovarian Symptoms and Treatment, a patient-reported outcome measure of symptom burden and impact of chemotherapy in recurrent ovarian cancer. *Quality Life Research* 27:59–74. 10.1007/s11136-017-1729-8 29248998

[19] Goldberg DP, Gater R, Sartorius N et al (1997) The validity of two versions of the GHQ in the WHO study of mental illness in general health care. Psychol Med 27:191–197 10.1017/s0033291796004242 9122299

[20] Goldberg D, Williams P (2000) General health questionnaire (GHQ). Nelson, Swindon, Wiltshire, UK

[21] Cella DF, Tulsky DS, Gray G et al (1993) The Functional Assessment of Cancer Therapy (FACT) Scale: development and validation of the general measure. J Clin Oncol 11(3):570–579 10.1200/JCO.1993.11.3.570 8445433

[22] Boyes A, Girgis A, Lecathelinais C (2009) Brief assessment of adult cancer patients’ perceived needs: development and validation of the 34-item Supportive Care Needs Survey (SCNS-SF34). J Eval Clin Pract 15(4):602–606 10.1111/j.1365-2753.2008.01057.x 19522727

[23] Foster C, Breckons M, Cotterell P et al (2015) Cancer survivors’ self-efficacy to self-manage in the year following primary treatment. J Cancer Surviv 9(1):11–19 10.1007/s11764-014-0384-0 PMC4341005 25028218

[24] Braun V, Clarke V (2006) Using thematic analysis in psychology. Qual Res Psychol 3(2):77–101

[25] Roland KB, Rodriguez JL, Patterson JR, Trivers KF (2013) A literature review of the social and psychological needs of ovarian cancer survivors. Psychooncology 22(11):2408–2418 10.1002/pon.3322 PMC11299102 23760742

[26] Cancer Council Victoria, Department of Health Victoria (2021) Optimal care pathway for women with ovarian cancer, 2nd edn. Cancer Council Victoria, Melbourne

[27] von Gruenigen VE, Huang HQ, Cella D et al (2018) Quality of life, symptoms and care needs in patients with persistent or recurrent platinum-resistant ovarian cancer: an NRG Oncology/Gynecologic Oncology Group study. Gynecol Oncol 150(1):119–126 10.1016/j.ygyno.2018.05.017 PMC6874212 29778506

[28] Rietveld MJA, Husson O, Vos MC et al (2018) Association between information provision and supportive care needs among ovarian cancer survivors: a cross-sectional study from the PROFILES registry. Psychooncology 27:1922–1929 10.1002/pon.4742 29683231

[29] Chan RJ, Knowles R, Ashbury FD et al (2024) Supportive care 2030 movement: towards unifying ambitions for global excellence in supportive cancer care - an international Delphi study. eClinicalMedicine 76:102825 10.1016/j.eclinm.2024.102825 PMC11415959 39309723

[30] Kwok C, Degen C, Moradi N, Stacey D (2022) Nurse-led telehealth interventions for symptom management in patients with cancer receiving systemic or radiation therapy: a systematic review and meta-analysis. Support Care Cancer 30(9):7119–7132 10.1007/s00520-022-07052-z PMC9008678 35420331

[31] Suh S-R, Lee MK (2017) Effects of nurse-led telephone-based supportive interventions for patients with cancer: a meta-analysis. Oncol Nurs Forum 44(4):e168–e184 10.1188/17.ONF.E168-E184 28632251

[32] Pradhan P, Sharman AR, Palme CE et al (2024) Models of survivorship care in patients with head and neck cancer in regional, rural, and remote areas: a systematic review. J Cancer Surviv. 10.1007/s11764-024-01643-x PMC12906516 39031309

[33] Zachou G, El-Khouly F, Dilley J (2023) Evaluation of follow-up strategies for women with epithelial ovarian cancer following completion of primary treatment. Cochrane Database Syst Rev 8(8):CD006119 10.1002/14651858.CD006119.pub4 PMC10471005 37650760

[34] Lanceley A, Berzuini C, Burnell M et al (2017) Ovarian cancer follow-up: a preliminary comparison of 2 approaches. Int J Gynecol Cancer 27(1):59–68 10.1097/IGC.0000000000000877 28002208

[35] Cox A, Bull E, Cockle-Hearne J et al (2008) Nurse led telephone follow up in ovarian cancer: a psychosocial perspective. Eur J Oncol Nurs 12(5):412–417 10.1016/j.ejon.2008.06.002 18845479

[36] Cohen PA, Webb PM, King M et al (2022) Getting the MOST out of follow-up: a randomized controlled trial comparing 3 monthly nurse led follow-up via telehealth, including monitoring CA125 and patient reported outcomes using the MOST (Measure of Ovarian Symptoms and Treatment concerns) with routine clinic based or telehealth follow-up, after completion of first line chemotherapy in patients with epithelial ovarian cancer. Int J Gynecol Cancer 32(4):560–565 10.1136/ijgc-2021-002999 34551895

[37] Cancer Australia (2023). Australian Cancer Plan (Summary), Cancer Australia, Surry Hills, NSW. Available from: https://www.australiancancerplan.gov.au/welcome. Accessed 24 Sep 2025.

