# Supplementary material for: Optimising ovarian cancer care: An embedded mixed methods evaluation of a national nurse-led telehealth program
Source: Support Care Cancer. 2026 Oct 7;34(10):1067. doi: 10.1007/s00520-026-11284-8 (PMC13642418; doi:10.1007/s00520-026-11284-8)
Supplement: Supplementary file 1 [file 520_2026_11284_MOESM1_ESM.docx]

**Supplementary 1 Interview Guide**

*Warm-up*

- Remind the participant that the interview will be recorded; if the participant does not wish for the interview to be recorded, let them know that they are ok with their choice and that we will not go ahead with the interview.
- Begin with some background questions about their current stage and treatment status.

*Main interview*

Concerns and experiences

- What have been your main concerns – either now or in the past? These could be medical, practical, family-related, or social concerns. For example, some people mention things like uncertainty, surgical menopause, sexuality or sexual functioning, relationships, or finances.
  - If there is anything you would rather not talk about, that is completely fine—just let me know.
- Thinking back to when you were first diagnosed, what bothered you the most at that time? And what about now – what is on your mind these days?

Coping and support

- How have you been coping with these challenges? Are you aware of any services and support available to you?
  - If yes, what services and support have you used or received? How did that experience feel for you?
  - If no, what services and support would you like to have?

Program experience

- How did you first hear about the Teal Support program? Can you remember your first interaction with the nurse from the program? What was that like for you?
  - In your opinion, when would be the best time for someone to learn about the program?
  - When the nurse called you, what kind of things did she usually talk about?
  - What was the reason you reach out to the nurse?

Value and improvements

- What has been the most valuable part of participating in the program or receiving support from the nurse? Why was that particularly meaningful to you?
- What about things that you think the program could be improved, or done differently to better support participants like yourself?

*Wrap up*

- Is there anything else that you would like to share about the program or your experience?

**Supplementary 2** **Associations between baseline patient-reported outcome measures (PROMs)**

| **Spearman’s correlation** | | MOST-Abdo | MOST-DorT | MOST-Chemo | MOST-Psych | MOST-Wellbeing | GHQ | FACT-PWB | FACT-SWB | FACT-EWB | FACT-FWB | FACT-OCS | SCNS-Physical | SCNS-Psycho | SCNS-Sexuality | SCNS-Care | SCNS-Infor | Self-efficacy |
| --- | --- | --- | --- | --- | --- | --- | --- | --- | --- | --- | --- | --- | --- | --- | --- | --- | --- | --- |
| **Symptoms & wellbeing (MOST) ^a^** | Abdominal (Abdo) | - | 0.734* | 0.422* | 0.374* | -0.367* | 0.374* | -0.636* | -0.373* | -0.323* | -0.441* | -0.491* | 0.518* | 0.411* | 0.316* | 0.309* | 0.342* | -0.515* |
|  | Disease/treatment-related (DorT) | - | - | 0.467* | 0.467* | -0.390* | 0.477* | -0.750* | -0.420* | -0.467* | -0.613* | -0.548* | 0.589* | 0.395* | 0.309* | 0.299* | 0.410* | -0.601* |
|  | Chemotherapy-related (Chemo) | - | - | - | 0.343* | -0.191 | 0.298* | -0.441* | -0.155 | -0.200* | -0.259* | -0.249* | 0.543* | 0.446* | 0.124 | 0.217* | 0.249* | -0.265* |
|  | Psychological (Psych) | - | - | - | - | -0.332* | 0.657* | -0.509* | -0.322* | -0.576* | -0.501* | -0.319* | 0.419* | 0.595* | 0.065 | 0.316* | 0.401* | -0.441* |
|  | Wellbeing (Wellbeing) | - | - | - | - | - | -0.364* | 0.408* | 0.337* | 0.244* | 0.379* | 0.288* | -0.321* | -0.348* | -0.141 | -0.257* | -0.387* | 0.516* |
| **Psychological (GHQ) ^b^** | Level of distress | - | - | - | - | - | - | -0.665* | -0.341* | -0.484* | -0.626* | -0.493* | 0.403* | 0.466* | 0.182 | 0.277* | 0.260* | -0.494* |
| **Quality of life (FACT) ^c^** | Physical wellbeing (PWB) | - | - | - | - | - | - | - | 0.330* | 0.445* | 0.675* | 0.533* | -0.619* | -0.433* | -0.292* | -0.188 | -0.286* | 0.602* |
|  | Social/Family wellbeing (SWB) | - | - | - | - | - | - | - | - | 0.328* | 0.438* | 0.438* | -0.266* | -0.215* | -0.229* | -0.298* | -0.311* | 0.437* |
|  | Emotional wellbeing (EWB) | - | - | - | - | - | - | - | - | - | 0.509* | 0.373* | -0.235* | -0.443* | -0.035 | -0.331* | -0.318* | 0.402* |
|  | Functional wellbeing (FWB) | - | - | - | - | - | - | - | - | - | - | 0.644* | -0.473* | -0.392* | -0.173 | -0.250* | -0.347* | 0.472* |
|  | Ovarian cancer specific concerns (OCS) | - | - | - | - | - | - | - | - | - | - | - | -0.310* | -0.261* | -0.074 | -0.183 | -0.244* | 0.421* |
| **Supportive care needs (SCNS) ^d^** | Physical & daily living | - | - | - | - | - | - | - | - | - | - | - | - | 0.523* | 0.262* | 0.326* | 0.413* | -0.442* |
|  | Psychological | - | - | - | - | - | - | - | - | - | - | - | - | - | 0.280* | 0.549* | 0.465* | -0.436* |
|  | Sexuality | - | - | - | - | - | - | - | - | - | - | - | - | - | - | 0.314* | 0.314* | -0.268* |
|  | Patient care & support | - | - | - | - | - | - | - | - | - | - | - | - | - | - | - | 0.639* | -0.396* |
|  | Health system & information | - | - | - | - | - | - | - | - | - | - | - | - | - | - | - | - | -0.443* |
| **Self-efficacy ^e^** | Total score | - | - | - | - | - | - | - | - | - | - | - | - | - | - | - | - | - |

**Note:** * p<0.05; Score range: ^a^ MOST 0-100; ^b^ GHQ 0-12; ^c^ PWB 0-28, SWB 0-28, EWB 0-24, FWB 0-28, OCS 0-44, FACT-O total 0-152; ^d^ Number of items reporting some need in each domain: physical and daily living 0-5, psychological 0-10, sexuality 0-3, patient care and support 0-5, health system and information 0-11; ^e^ 1-10.

**Supplementary 3 Associations between participants’ utilisation of TSP services and their baseline outcome measures (PROMs)**

| **Spearman’s correlation** | | Total TSP services | Information support | Ongoing support | Care coordination | Psychosocial support | Crisis support | Other purposes |
| --- | --- | --- | --- | --- | --- | --- | --- | --- |
| **Symptoms & wellbeing (MOST) ^a^** | Abdominal (Abdo) | 0.118 | 0.125 | 0.095 | 0.157 | 0.049 | 0.097 | -0.201 |
|  | Disease/treatment-related (DorT) | 0.241* | 0.146 | 0.223* | 0.329* | 0.010 | 0.035 | -0.177 |
|  | Chemotherapy-related (Chemo) | 0.008 | 0.062 | 0.017 | 0.170 | -0.021 | -0.206 | -0.234 |
|  | Psychological (Psych) | 0.278* | 0.176 | 0.191 | 0.276 | -0.030 | -0.076 | 0.012 |
|  | Wellbeing (Wellbeing) | -0.363* | -0.249* | -0.343* | -0.125 | -0.294 | -0.243 | -0.388* |
| **Psychological (GHQ) ^b^** | Level of distress | 0.246* | 0.070 | 0.200 | 0.430* | 0.028 | -0.108 | -0.180 |
| **Quality of life (FACT) ^c^** | Physical wellbeing (PWB) | -0.215* | -0.157 | -0.172 | -0.342* | -0.043 | -0.103 | 0.237 |
|  | Social/Family wellbeing (SWB) | -0.230* | 0.012 | -0.236* | -0.095 | -0.008 | -0.371* | -0.316 |
|  | Emotional wellbeing (EWB) | -0.197 | 0.002 | -0.195 | -0.047 | 0.102 | 0.184 | 0.182 |
|  | Functional wellbeing (FWB) | -0.234* | -0.063 | -0.266* | -0.245 | -0.012 | 0.041 | 0.112 |
|  | Ovarian cancer specific concerns (OCS) | -0.289* | -0.039 | -0.272* | -0.345* | 0.011 | -0.030 | 0.189 |
| **Supportive care needs (SCNS) ^d^** | Physical & daily living | 0.131 | 0.117 | 0.146 | 0.181 | 0.128 | -0.067 | -0.115 |
|  | Psychological | 0.156 | 0.012 | 0.151 | 0.110 | 0.185 | 0.023 | -0.104 |
|  | Sexuality | -0.085 | -0.181 | -0.068 | 0.200 | 0.007 | 0.216 | -0.042 |
|  | Patient care & support | 0.073 | 0.045 | 0.029 | 0.149 | 0.171 | -0.169 | -0.214 |
|  | Health system & information | 0.167 | 0.150 | 0.137 | -0.068 | 0.263 | -0.070 | -0.133 |
| **Self-efficacy ^e^** | Total score | -0.272* | -0.126 | -0.263* | -0.097 | -0.131 | -0.253 | 0.035 |

**Note:** * p<0.05; Score range: ^a^ MOST 0-100; ^b^ GHQ 0-12; ^c^ PWB 0-28, SWB 0-28, EWB 0-24, FWB 0-28, OCS 0-44, FACT-O total 0-152; ^d^ Number of items reporting some need in each domain: physical and daily living 0-5, psychological 0-10, sexuality 0-3, patient care and support 0-5, health system and information 0-11; ^e^ 1-10.
